# Case of Fungus Hæmatodes

**Published:** 1848-02

**Authors:** John G. Meachem

**Affiliations:** Linden, Genesee Co., N.Y.


ART. II.—*Case of Fungus Hæmatodes.* Communicated by John G. Meachem, M. D.

Linden, Genesee Co., N. Y., Dec. 15, 1847.

Dear Sir:—If you think the following case of sufficient interest to merit a place in your Journal, it is at your disposal:—

*Fungus Hæmatodes.*—Mrs. V——, aged 49, in the begining of January, 1847, complained of slight lancinating pains in her right breast, which extended into the axilla, and up along the neck. A few days after, on examination, a tumor was detected about two inches outside of the nipple, and deep in the substance of the breast, which has continued to increase, attended with severe pain, until now it is much larger than a man's hat, weighing, I presume, twenty-five or thirty pounds; of a deep purple color, having on its end a fungus protuberance as large as a full grown fœtal head. It has been soft and elastic from the commencement, leading some to suppose that it contained pus, consequently, poultices, &c,, have been applied with a view of bringing it to an head. In October three or four prominences appeared on its surface, two of which increased until they developed the charactersof fungoid excrescence, showing to the incredulous its fatal nature.' For some time previous to the appearance of the fungus, a copious discharge of yellow serum issued from the nipple, and, since that time, from the fungus as, also, occasionally considerable blood. The constitution has become deeply implicated, and it is more than probable that a few days will terminate her earthly existence.

The first treatment pursued in this case, was the free use of iodine, internally and externally; but the patient and her friends, being less enlightened on medical subjects than many people, you may suppose, consulted more than one physician, as well as some that were not physicians properly, such as, "*celebrated cancer doctors.*"

The necessity of extirpation, as the only means of prolonging life, was, from time to time, urged upon her, but fear of the knife, and a hope, induced and sustained by pretenders, that it might not prove fatal, have served to intimidate a mind naturally undecided.

JOHN G. MEACHEM, M. D.

Austin Flint, M. D.

